# Probing the Luminal Compartment of 3D Organoids via Particle Tracking Microrheology

**DOI:** 10.21769/BioProtoc.5830

**Published:** 2026-09-20

**Authors:** Katrina N. Lyon, Barkan Sidar, Cameron Dudiak, Grace Jordan, Diane Bimczok

**Affiliations:** 1 Department of Neurology, University of Colorado Anschutz Medical Campus, Aurora, CO, USA; 2 Department of Microbiology and Cell Biology, Montana State University, Bozeman, MT, USA; 3 Chemical and Biological Engineering Department, Montana State University, Bozeman, MT, USA; 4 Center for Biofilm Engineering, Montana State University, Bozeman, MT, USA; 5 Department of Mathematical Sciences, Montana State University, Bozeman, MT, USA

**Keywords:** Organoid, Mucus, Microrheology, Particle tracking, Rheology

## Abstract

The mucus layer lining the human stomach is a critical barrier that protects the underlying epithelium from gastric acid and harmful pathogens such as *Helicobacter pylori*. The efficacy of this barrier relies on the structural integrity of the mucus, which is determined by various biochemical and biophysical features. Human gastric organoids—3D cellular models that resemble the stomach—contain mucus and have been used to investigate gastric disease. The luminal compartment of three-dimensional epithelial organoids represents a physiologically relevant but experimentally inaccessible microenvironment. In gastric organoids, luminal accumulation of mucus creates a confined viscoelastic hydrogel that mimics native gastric mucus. However, the small volume and topological confinement of organoids preclude conventional bulk rheometry. Here, we describe a particle tracking microrheology (PTM) protocol to measure the viscoelastic properties of the mucus within intact organoid lumina following microinjection of fluorescent microspheres. High-speed fluorescence imaging and particle trajectory analysis enable the quantification of viscous and elastic properties of the mucus through calculation of mean squared displacement (MSD), diffusive scaling exponent (alpha), and frequency-dependent storage (G’) and loss (G’’) moduli. This method enables rheological measurements in nanoliter-scale compartments without disrupting organoid architecture. We further discuss the impact of mucus heterogeneity and microstructure on scale-dependent mechanical behavior. This protocol is broadly applicable to other organoid systems and can be adapted to Transwell or organ-on-chip platforms for in situ luminal measurements.

Key features

• Enables rheological measurements in small (nanoliter scale) volumes.

• Compatible with intact, Matrigel-embedded 3D organoids.

• Allows in situ measurement without mucus harvest or purification.

• Resolves microscale heterogeneity inaccessible to bulk rheometry and is compatible with functional screening of mucus-modifying drugs.

## Graphical overview


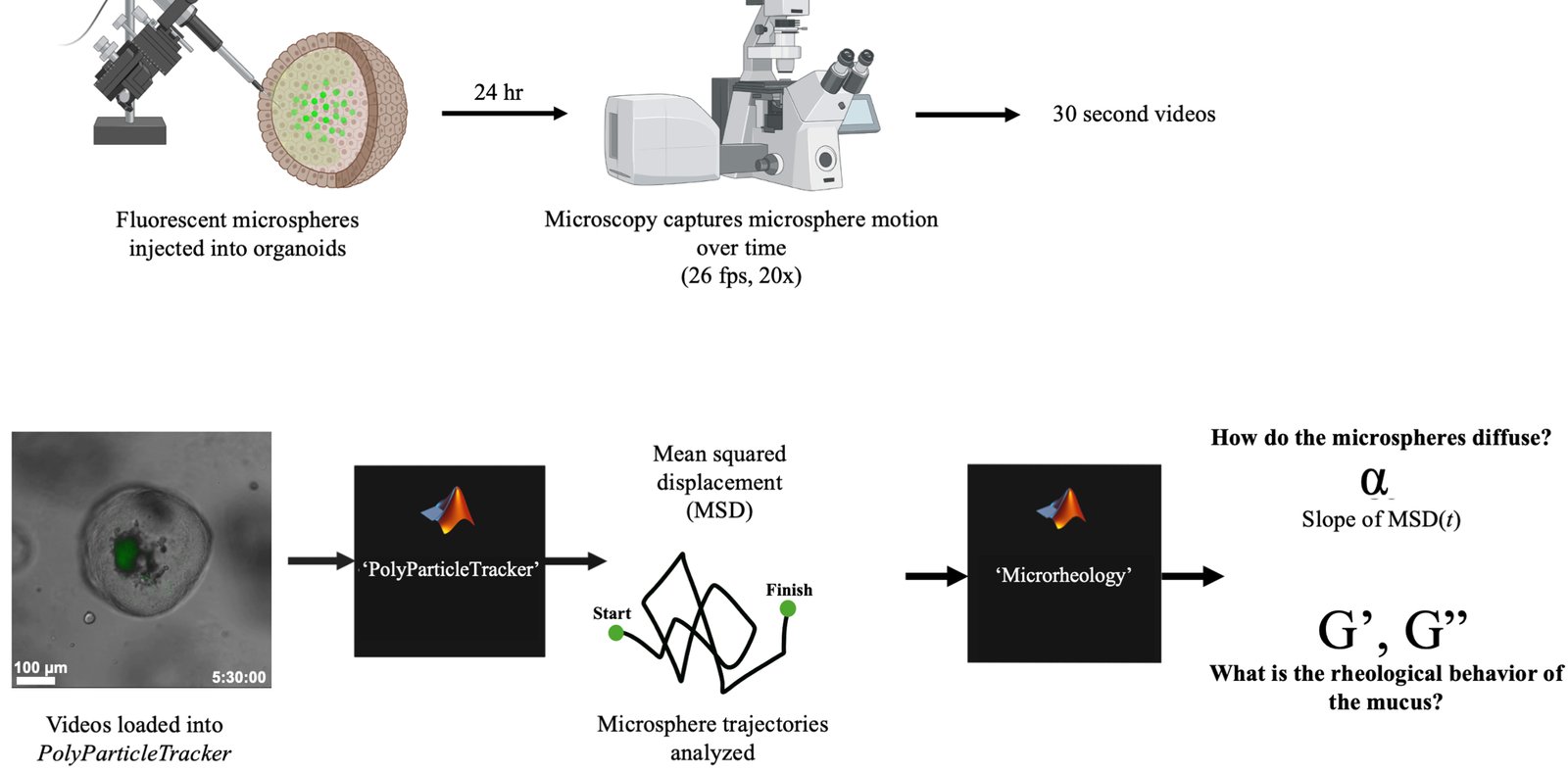


**Particle tracking microrheology in gastric organoids.** (Top) Fluorescent microspheres are injected into the organoids. After a 24-h equilibration period, timelapse confocal microscopy (20×) is used to capture 30-s videos at 26 frames per second. (Bottom) Using the PolyParticleTracker routine in MATLAB [1], videos are analyzed for mean squared displacement.

## Background

Organoids have significantly improved our ability to study tissue development and disease progression in vitro and provide a viable alternative to animal models for pre-clinical drug testing [2–4]. Gastrointestinal organoids are widely used to represent the stomach, small intestine, and colon in studies of gut health and disease [4–8]. Notably, such organoids are known to produce the protective mucus that lines the gastrointestinal tract [9–11]. Mucus is a viscoelastic material that plays an important role as the body’s first barrier against the outside world [12].

Human gastric organoids (HGOs) generally consist of the apical epithelium facing inward, separated from the outer environment [9,13]. HGOs contain both types of mucus-producing cells—MUC5AC-secreting pit mucus cells and MUC6-secreting mucus neck cells—that are present in the healthy human stomach and secrete mucus into the organoid lumen, as shown in multiple studies [5,14,15]. The topologically closed structure of HGOs makes the lumen challenging to access [12]. Investigating the suitability of HGO models for recreating in vivo gastric conditions—such as the protective mucus barrier—requires thorough characterization of their luminal composition. While fixing and sectioning organoids using histological methods can reveal a cross-section of the lumen, this process disrupts the natural state and spatial distribution of luminal contents [15]. Studying the luminal microenvironment within an organoid and the biophysical properties of its contents provides the most accurate reflection of its natural spatial complexity and minimizes experimental artifacts [16]. We and others have used micromanipulator-controlled microinjection to access the lumen of viable gastric organoids for delivering bacteria or fluorescent tracers [6,11,15,17,18]. Additionally, we have used microelectrodes to measure intraluminal oxygen content for bacterial inoculation [6] and pH [19], upon which mucus rheology is dependent. This non-destructive technique is powerful and has enabled real-time observations to be made, including that the organoid lumen maintains microaerophilic conditions and a near-neutral pH. In this study, fluorescent microspheres were microinjected into gastric organoids for passive particle tracking microrheology analysis, enabling the rheological characterization of the mucus within the organoids.

Previous work using microparticle tracking and oscillatory shear rheology has established the micro- and bulk rheological properties of mucus isolated from the stomach and other organs, as well as of several purified mucins from the lung, salivary, gastric, and reproductive systems [20–23]. These studies concluded that gastric mucins and mucus exhibit both viscous and elastic properties, with certain types having the ability to form elastic gels at low pH [24]. Many soft materials exhibit a viscoelastic response to applied shear forces that depends on how liquid-like (viscous) or solid-like (elastic) the material behaves [25]. More specifically, viscosity refers to a material’s ability to dissipate energy and resist flow, while elasticity refers to a material’s ability to store energy and return to its original shape following deformation [26,27]. The extent of each can depend upon the various forces acting on the material. Understanding the rheological behavior of gastric mucus may have physiological relevance to questions such as the response of mucus to shear forces associated with digestion [28] and the transport of nutrients and microorganisms across the mucus barrier [29].

In this protocol, gastric organoid luminal mucus is characterized by visualization of microinjected fluorescent microspheres into the lumen for particle tracking microrheology (PTM) using microscopy. We employ analytical methods to interrogate the thermally driven, Brownian motion of the injected microspheres to extract information about the mechanical properties of the luminal material [30]. We confirm that the mucus secreted by the organoids is viscoelastic, and that the viscoelasticity varies temporally, suggesting that gastric organoids can serve as a physiologically relevant model system for investigating the influence of other factors on mucus structure and dynamics [20,27,31,32]. Note that, throughout this protocol, we utilize the term *microspheres* to refer to the fluorescent polystyrene probes (e.g., 1.0 μm Fluoresbrite) injected into the gastric organoid lumen. However, it should be noted that in the broader microrheology literature, these probes can be referred to interchangeably as *beads, particles*, or *microparticles* [33,34].

## Materials and reagents

**Biological materials**

1. Human gastric organoids derived from adult stem cells

**Reagents**

1. Dulbecco’s phosphate-buffered saline (PBS) (without Ca^++^ and Mg^++^) (HyClone, catalog number: SH30028.03)

2. 70% ethanol (Fisher Bioreagents, catalog number: BP82031GAL)

3. Advanced DMEM/F-12 (Gibco, catalog number: 12-491-015)

4. Dulbecco’s modified Eagle medium (DMEM) (Fisher Scientific, catalog number: 15017CV)

5. Fetal bovine serum (HyClone Laboratories, catalog number: SH30088)

6. Gentamycin sulfate (IBI Scientific, catalog number: IB02030)

7. HEPES free acid (Cytiva, catalog number: SH30237.01)

8. L-glutamine (Cytiva, catalog number: SH3003401)

9. L-WRN cell culture supernatant (ATCC, catalog number: CRL-3276)

10. Fluoresbrite^®^ Yellow Green 1 μm polystyrene microspheres (Polysciences, catalog number: 17154-10)

11. Food coloring, blue (McCormick, catalog number: 43217-41014)

12. Y27632 (Tocris, catalog number: 1254)

13. SB431542 (Tocris, catalog number: 1614)

14. Amphotericin B (Fungizone) (HyClone Laboratories, catalog number: SV30078.01)

15. Trypsin-EDTA 0.025%, phenol red (Gibco, catalog number: 25-200-056)

16. Matrigel Membrane Matrix 354234 (Corning, catalog number: CB-40234)

17. Penicillin/Streptomycin (10,000 U/ mL) (Gibco, catalog number: 15-140-148)

18. Collagenase type IV (Sigma, catalog number: C5138-5G)

**Solutions**

1. Organoid expansion media (see Recipes)

**Recipes**

**1. Organoid expansion media**

**Table:** 

Reagent	Concentration
L-WRN cell culture supernatant	50%
Advanced DMEM/F12	37%
Fetal bovine serum	10%
Penicillin/streptomycin	100 U/mL
L-glutamine	2 mM
Gentamycin	50 μg/mL
Amphotericin B	0.25 μg/mL
HEPES buffer	10 mM
Y-27632	10 μM
SB431542	10 μM

*Note: The media used to culture organoids may vary by cell type and research group preference.*

**Laboratory supplies**

1. 35 mm dish with no. 1.5 coverslip (MatTek, catalog number: P35G-1.5-20-C)

2. 5 mL serological pipette, individually wrapped, paper/plastic, bag, sterile (CellTreat, catalog number: 229091B)

3. 10 mL serological pipette, individually wrapped, paper/plastic, bag, sterile (CellTreat, catalog number: 229092B)

4. 25 mL serological pipette, individually wrapped, paper/plastic, bag, sterile (CellTreat, catalog number: 229093B)

5. 15 mL centrifuge tube-foam rack, sterile (CellTreat, catalog number: 229412)

6. 50 mL centrifuge tube-foam rack, sterile (CellTreat, catalog number: 229422)

7. 24-well tissue culture plate, sterile (CellTreat, catalog number: 229124)

8. 70 μm cell strainer, individually wrapped, sterile (CellTreat, catalog number: 229483)

9. 1,000 μL extended length low retention pipette tips, racked, sterile (CellTreat, catalog number: 229037)

10. Glass capillary tubes (3.5” long) (Drummond, catalog number: 3-000-203-G/X)

## Equipment

1. Nanoject-II (Drummond, catalog number: 3-000-204)

2. MM33 right-handed micromanipulator (Marzhauser Wetzlar, catalog number: 61-42-113-0000)

3. Stereomicroscope (e.g., Fisher Science Education, model: 430TBL)

4. Leica SP5 CLSM (Leica) or similar confocal laser scanning microscope set up on a floating microscope (air) table

5. Environmental control imaging chamber (Life Imaging Services)

6. Biosafety Cabinet Class II Type A/B3 (Nuaire, catalog number: NU-425-600)

7. Incubator (Fisher Scientific, catalog number: 11676604)

8. Micropipette puller Model P-87 (Sutter Instrument Co.)

## Software and datasets

1. MATLAB [MathWorks, v7.9.0.529 (R2009b)]; package provided in supplemental data for Rogers et al. 2007 [1]; a complete list of applicable MATLAB packages for this protocol is provided in the supplementary data

2. PolyParticleTracker (https://iopscience.iop.org/article/10.1088/1478-3975/4/3/008/data)

*Note: PolyParticleTracker requires a MATLAB version between V7.0 and V2009.b.*

3. Leica LASX Version 5 or newer, or other applicable microscope software

## Procedure

*Note: It is highly recommended to perform periodic validation of the particle tracking analysis pipeline using Newtonian fluids such as water (as a negative control) or glycerol (as a positive control for subdiffusive rheological behavior). Such control measurements may also be conducted before attempting the protocol with organoids, to ensure that the user’s particular microscope settings (such as frame rate) are sufficient to capture the desired information.*

**A. Preparation of organoids for microinjection**

*Notes:*

*1. The protocol for organoid generation and culture is provided as an example. The PTM protocol is expected to work with any spherical organoids of similar size. Please refer to other published protocols for more details on general organoid culture methods (e.g., [2,13,35]).*

*2. Organoids for our experiments are derived from gastric gland preparations, which are obtained from adult human tissue as described above. We use a collagenase tissue digestion method to isolate these glands before they are suspended in extracellular matrix, as previously described [5,6,11,36,37]. Alternative protocols for organoid generation and maintenance may be equally suitable [2,5,38].*

1. Isolate gastric glands from stomach tissue by digestion with 0.5 U/mL collagenase type IV for 1 h at 37 °C and 200 rpm [9]. Pellet glands and embed the suspension into Matrigel at a 1:4 ratio. Allow Matrigel to polymerize for 15–20 min at 37 °C and overlay Matrigel containing the glands with gastric organoid expansion medium. Organoids should be ready for passage in 5–7 days, with media changes every other day.

2. Maintain gastric organoids in 24-well plates using 500 μL of expansion media per well [3,5,32]. Replenish media every other day and passage organoids every 5–7 days. For particle tracking microrheology (PTM) experiments, organoids below passage 15 will be seeded in a MatTek 35 mm glass-bottom dish.

3. While preparing organoids for transfer to a 35 mm glass-bottom dish, allow Matrigel aliquot(s) to thaw on ice for at least 45 min. Maintain Matrigel on ice throughout this protocol to prevent premature gelation. Prewarm cell culture plates by placing them in a 37 °C, 5% CO_2_ incubator.

4. Remove the media from each well and harvest the gastric organoid cultures by pipetting ice-cold PBS onto each Matrigel droplet and scratching the gel with the tip of a P1000 pipette. Pipette the PBS with the Matrigel fragments containing organoids into a 15 mL conical tube.

5. Centrifuge the tube at 200× *g* for 5 min at 4 °C. Matrigel fragments containing the organoids will be visible as a compact layer at the bottom of the tube. Carefully aspirate the supernatant and pipette 350 μL of 0.25% Trypsin-EDTA into each tube, mixing gently by pipetting up and down. Incubate the tubes in a 37 °C water bath for 2–5 min.

6. Following incubation with trypsin-EDTA, add 600 μL of ice-cold DMEM with penicillin/streptomycin to each tube and pipette up and down at least 40× to break the organoids into fragments. Centrifuge at 200× *g* for 5 min at 4 °C. Aspirate and discard the supernatant. To set up cultures for injection, resuspend the cell pellet in ice-cold, liquid Matrigel at a 1:4 v/v ratio of organoid pellet to Matrigel for plating.

7. For each sample, plate 40 μL of liquid Matrigel containing organoids/organoid fragments in a thin horizontal line along the diameter of a 35 mm glass-bottom dish.

*Note: Dispensing the gel onto the plate in a line instead of a round droplet thins out the culture, enabling easier access to individual organoids for injection and easier visualization of individual organoids.*

**Figure 1.**
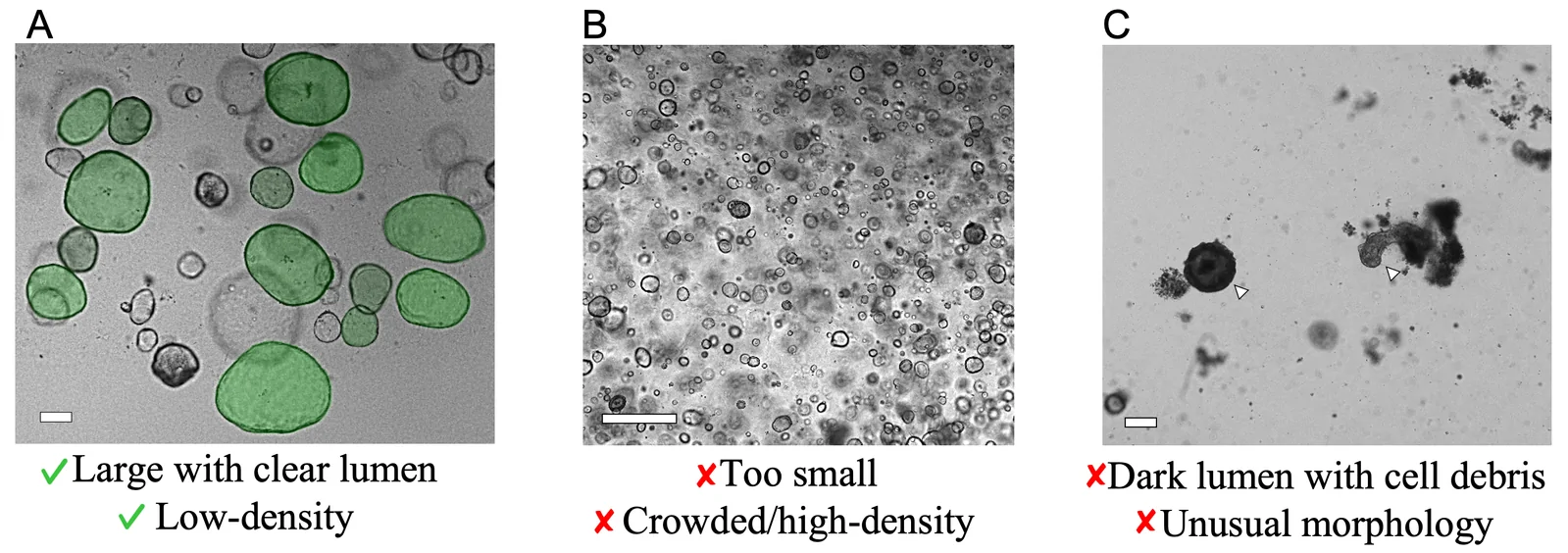
Suitability of an organoid culture for microinjection. (A) Example of a culture in which organoids are roughly between 200 and 800 μm with plenty of space to navigate between them. The lumen of an ideal organoid is easily distinguishable from its thin surrounding epithelial border. Organoids highlighted in green represent those that the authors would select for injection (n ≥ 10). Notably, there are also several good candidate organoids in another focal plane that are still accessible and should be considered for injection as well. (B) Example of a culture in which the organoids are both too small to easily inject and too crowded to navigate from one to the next. (C) Arrows indicate an example of an organoid (left) with a dark lumen (indicating cell death and debris) and an organoid with unusual morphology (right). It is recommended to use cultures in which organoids are more uniformly spherical, as complex morphologies can render the lumen more difficult to access and subsequently inject into. All scale bars correspond to 200 μm.

8. Allow the Matrigel to polymerize for 15–30 min, then carefully add 2 mL of organoid expansion media to the plate by pipetting along the edge of the plate to avoid disturbing the Matrigel.

9. Replace the media every other day. Allow 4–8 days for a minimum of 10 organoids to grow to a minimum diameter of 400 μm.

10. To proceed with microinjection, ensure that each culture contains at least 10 organoids that meet the following criteria: >200 μm, healthy appearance (little to no dark material visible within the lumen via brightfield microscopy), and not directly adjacent to other organoids (see Figure 1). It is recommended, whenever possible, to inject organoids closer to the glass bottom of the culture dish. This increases the likelihood that the injected organoids are within the working distance of the microscope user’s chosen objective.

**B. Preparation of microsphere suspension**

1. Vortex the stock bottle for ~15 s at 2,500–3,000 rpm and dilute approximately 1:1,000 to a final concentration of approximately 4.55 × 10^7^ microspheres/mL in sterile PBS.

*Note: A visual spot check under a microscope may be helpful in determining whether any microsphere clusters have broken up or if further vortexing may be necessary.*

2. Add a trace amount (no more than 10 μL) of food coloring dye to the microsphere suspension for visualization of successful injection.

*Note: Suspension can be stored either at room temperature or at 4 °C prior to beginning the next section.*

**C. Microinjection**

1. Use a micropipette puller with air pressure set to 200 (an arbitrary, unitless value here) to pull a glass capillary into a needle shape. This will create two injection capillaries once cut in half. At this point, the capillary tips are still closed.

2. To open the capillary tip, gently tape the far end to a microscope slide and place it under a stereomicroscope. Adjust the focus on the microscope until the needle end of the capillary is clearly visible. Using one swift rocking motion, roll a scalpel blade perpendicularly over the needle, approximately 0.5–1.0 mm from the tip. This will create an opening, and the capillary is ready for use.

3. Backfill a 2 μL glass capillary with sterile mineral oil, load it onto a micromanipulator-controlled nanoliter autoinjector (Nanoject II), and fill it with the microsphere suspension.

*Note: Briefly vortex the microsphere suspension and pipette a drop onto a slide or 35 mm dish. Load the capillary from this droplet. Trying to maneuver the capillary into a microcentrifuge tube could result in damage to the tip.*

4. Position the injection capillary so that it is prepared to advance toward the first organoid of interest along the axis perpendicular to the epithelial surface. Allow the capillary tip to make a minor indentation on the surface without penetrating.

5. With one swift motion, advance the capillary into the organoid.

6. Inject at least 10 organoids of at least 200 μm in diameter with 9.2 nL of the suspension [39].

**Critical:** Organoids with weaker structural integrity, usually those greater than 1 mm in diameter, may collapse when penetrated with the capillary [20].

7. Return organoids to the 37 °C, 5% CO_2_ incubator for 24 h prior to imaging to allow equilibration of the system.

**Critical:** We previously showed that 30%–35% of organoids may spontaneously rupture over a 24-h period [9]. In the dataset shown here, 1 in 10 organoids were excluded due to rupture. Expelled luminal contents will be visible in the Matrigel immediately surrounding the ruptured organoid. Organoids that have ruptured can still retain some fluorescent microspheres but may provide results that are difficult to interpret and should therefore be excluded from the analysis.

Tip: Inject more organoids than required for imaging, with the expectation that such rupture events may occur.

**D. Live imaging**

**Figure 2.**
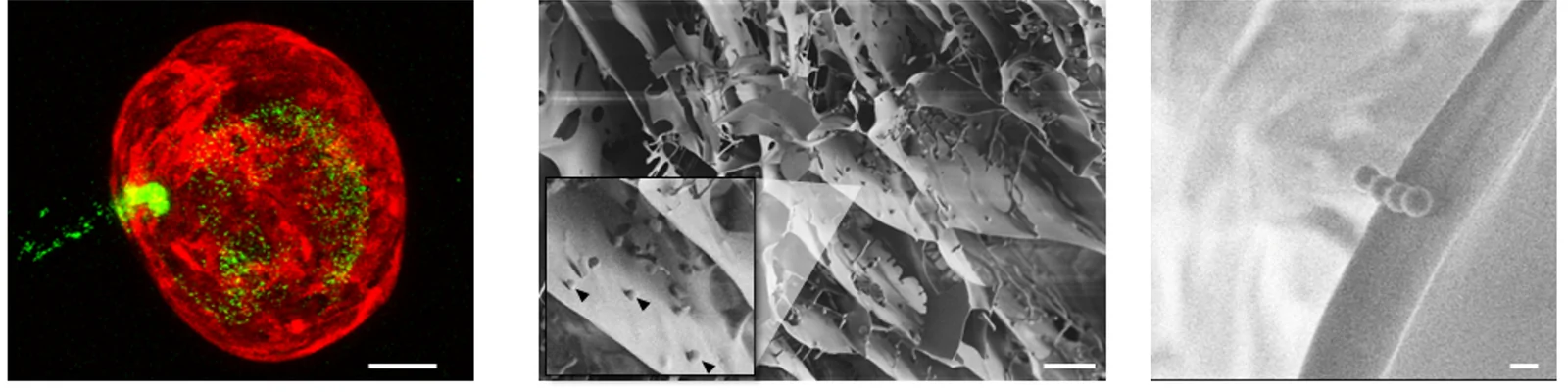
Confocal and cryo-field emission scanning electron microscopy images of microspheres in organoid mucus. (Left) Fluorescent particles (green) in the lumen of an organoid (red) expressing mCherry following lentiviral transduction. Scale bar = 100 μm. (Middle) CryoFE-SEM image of particles (indicated by black arrows) resting around honeycomb scaffolds in mucus samples apically secreted by organoid-derived air–liquid interface cultures. Scale bars = 10 μm (left) and 1 μm (Right).

1. Image organoids on a confocal laser scanning microscope (CLSM, e.g., Leica SP5 or similar) on a heated stage with an environmental control chamber (37 °C, 5% CO_2_; Life Imaging Services). To prevent microsphere movement due to microscope vibrations, an air table microscope setup is ideal. Use a 20× (0.70 NA) objective with a 4× optical zoom or equivalent settings for particle tracking, capturing an average of 15 ± 5 trackable microspheres in 30-s videos at 26 frames per second (Figure 2A, Video 1).

*Notes:*

*1. “Trackable microspheres” refers to those with an area of at least 6 pixels [1].*

*2. If a comparison of rheological properties at different time points is desired, time-lapse imaging can be performed to confirm that organoids have not ruptured between time points. For our purposes, 10 organoids were imaged over 72 h at 5-min intervals.*

**Video 1.**
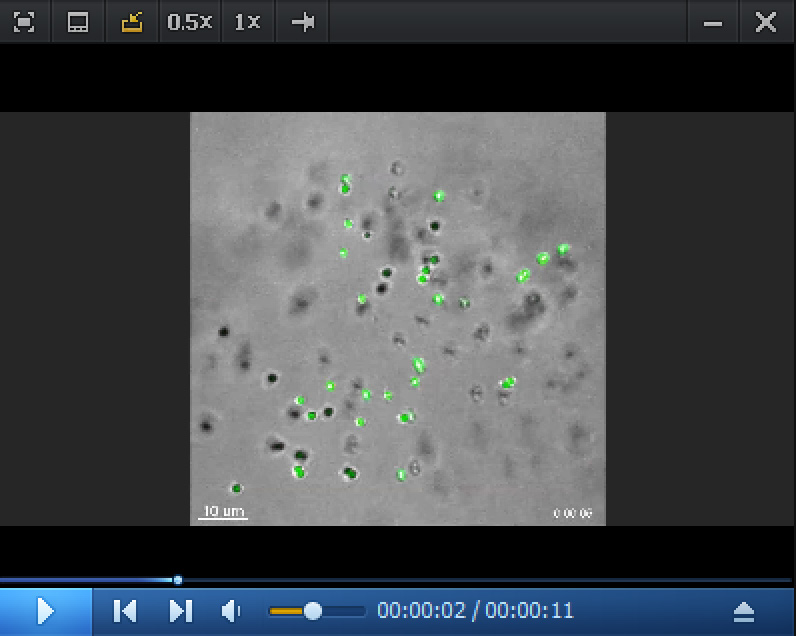
Motion of fluorescent microspheres inside the gastric organoid lumen

**E. Particle tracking analysis**

**Figure 3.**
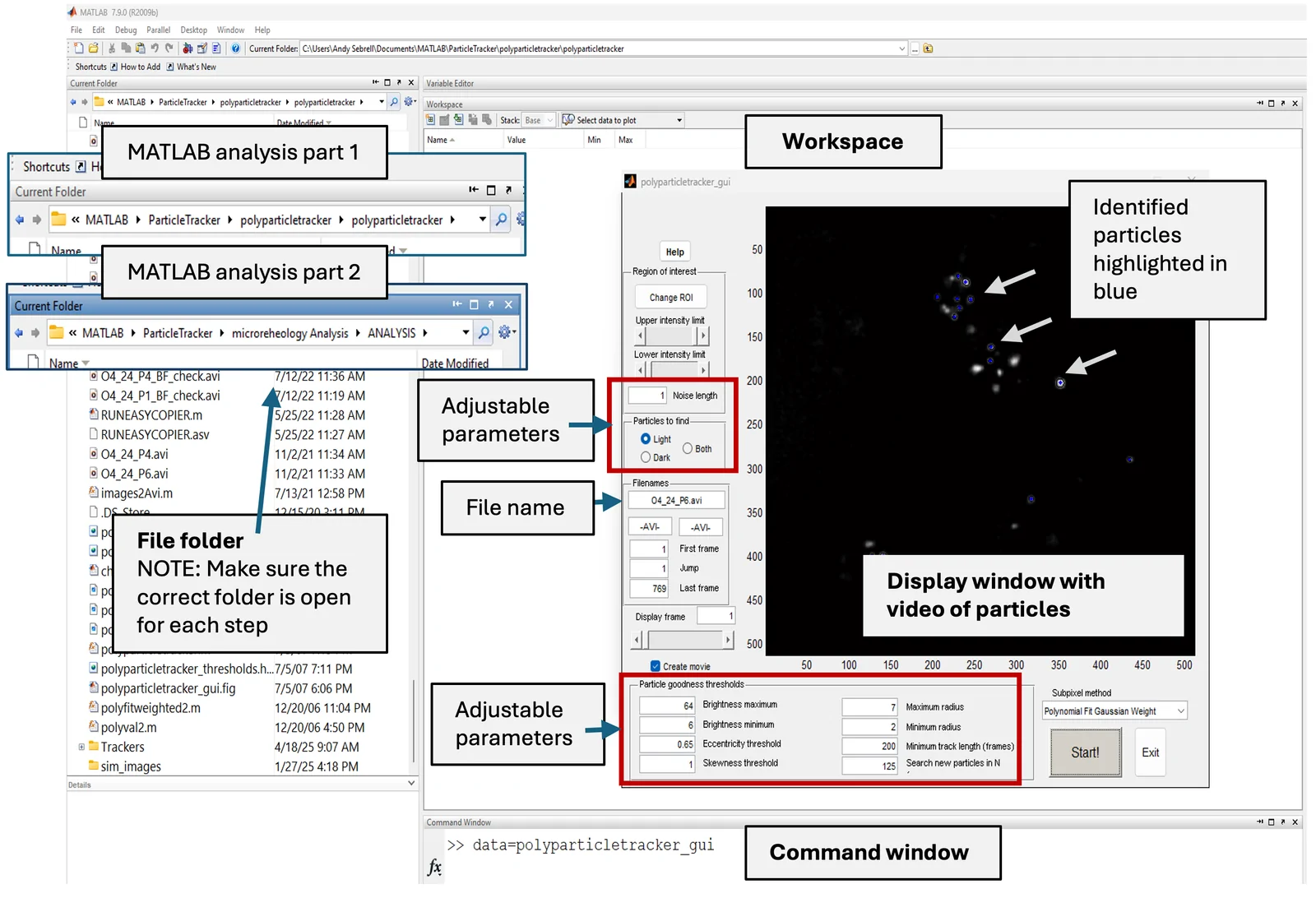
Overview of MATLAB particle tracker software

1. Confirm that the uploaded video is free of imaging artifacts (i.e., uneven illumination, excessive cellular debris, autofluorescence) and that there are at least 10–20 trackable microspheres in frame (see Data analysis section for more quality control criteria). If imaging was performed in the absence of an air table, drift correction can be applied to captured video files in FIJI/ImageJ. Under *Plugins*, select *Correct 3D Drift* and check boxes for *multi time scale computation* and *sub pixel drift correction*.

2. Save all video files as *.avi files with no compression.

3. Open MATLAB (see Figure 3) and open the folder with the saved AVIs.

**E1. MATLAB analysis part 1: polyparticletracker routine**

1. Copy the name of the first file to be analyzed, type the following into the command window, and hit Enter:

>> data = polyparticletracker_gui

2. In the pop-up window, paste the file name under *Prefix*.

3. Click into the display window (see Figure 3) to open the video.

4. Ensure the parameters are optimally established for your experiment and application. Our parameters are provided as an example:

a. Noise length: 1

b. Particles to find: Light

c. Brightness maximum: 64

d. Brightness minimum: 6

e. Eccentricity threshold: 0.65

f. Skewness threshold: 1

g. Maximum radius: 7

h. Minimum radius: 2

i. Minimum track length (frames): 200

j. Search new particles in N: 125

k. Subpixel method: polynomial fit Gaussian weight

*Note: For more information on each of these parameters, click the* Help *button in the pop-up window or refer to [1].*

Tip: Be sure to visually compare the particles selected in blue (see Figure 3) to the original videos and adjust settings if needed.

5. Hit *Start!* to begin analysis.

6. When the command window says *Done!*, click *Exit* to save data and type the following into the command window:

>>data = data.tr

7. Right-click the yellow data box in the workspace and save in ParticleTracker > microrheology Analysis > ANALYSIS. Paste the original file name but replace .avi with _yourname.mat. Repeat section E1 for all videos of interest before moving on.

**E2. MATLAB analysis part 2: ParticleTracker/microrheology analysis routine**

1. In the *Current Folder* window of MATLAB, go back to ParticleTracker, then move to where you saved the video data (microrheology Analysis > ANALYSIS).

2. Open microrheology_analysis_01_01_22 and check imaging parameters:

a. Temperature is 22 °C, 295 K

b. Radius of the beads (0.5 for 1-μm diameter beads)

c. Pixel size (based on original imaging parameters)

3. Copy the name of the file to be analyzed.

4. Right-click the file “microrheologyanalysis_01_01–22.m” and click *run file*, pasting the file name (in single quotations) into the MATLAB Command Window when prompted, as shown below. Hit Enter.

data file name = ? (eg. water.mat)

‘filename_yourname.mat’

**Figure 4.**
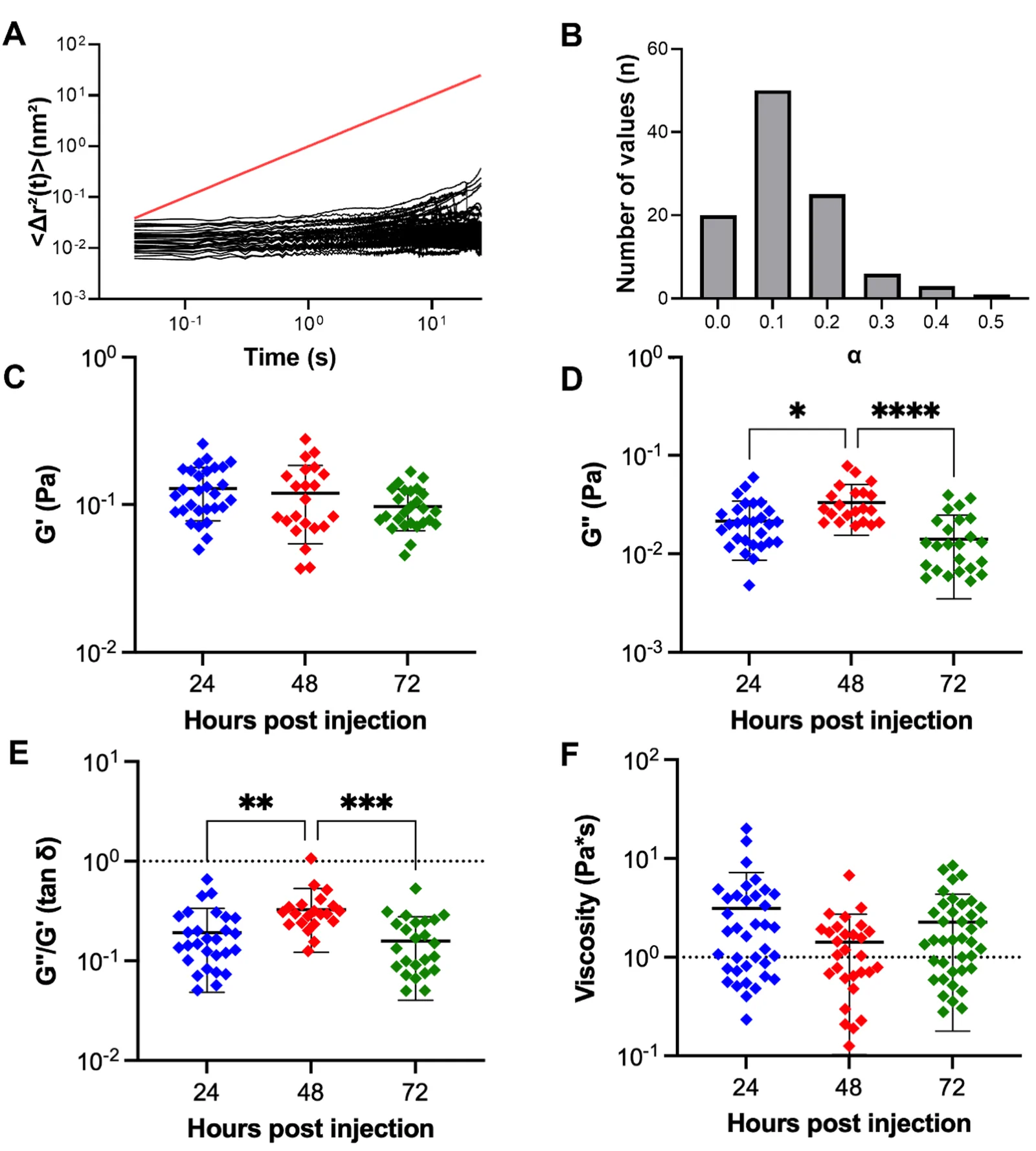
Particle-tracking microrheology reveals viscoelastic behavior of organoid luminal mucus. (A) Time-dependent mean-squared displacements (MSDs) of individual colloidal probe particles (n = 29; d = 1.0 μm) in the lumen of one representative organoid at 24 h post-injection. The red line is a reference corresponding to the expected MSD for particle motion in water (α = 1). (B) Distribution of α values for corresponding particle MSDs from all time points. (C) Frequency-dependent storage moduli (G’) and (D) loss moduli (G’’) of particles at 24 h (n = 29), 48 h (n = 22), and 72 h (n = 26) in the organoid lumen (ω = 1 Hz). Kruskal–Wallis test: *p = 0.043; **** p < 0.0001. (E) Ratio of G’’/G’ (tanδ), where >1 is viscous, and <1 is elastic. Kruskal–Wallis test: **p = 0.0037; ***p = 0.0003. (F) Viscosity of luminal mucus at ω = 1 Hz.

5. For “i = ?,” type 1 and hit enter.

6. When the program has finished running, a graph will appear, similar to that in Figure 4A. Exit out of the graph.

7. Go to MMM in the workspace and right-click to save the “MMM” file to a designated outputs folder, changing the name to add “_NEWFILENAME” before “.mat.”

8. Open the output folder from the previous step in the *Current Folder* window and run the file “everything2TEX_GOOD.m.”

9. Enter the name of the file you just saved in single quotes and hit Enter.

10. Rename the file, removing “_NEWFILENAME.” Type in single quotes and enter. Now, your data are saved in the TEX2 folder. This folder will contain data for G’, G’’, G’’/G’, and viscosity (Figure 4B–F).

*Note: The storage (G’) and loss (G’’) moduli refer, respectively, to a material’s energy stored and energy dissipated during deformation. These values can be interpreted as indicators of a material’s elastic (G’) or viscous (G’’) behavior. The generalized Stokes–Einstein equation uses the measured displacement of tracked particles to determine the storage and loss moduli of a material [40].*

11. Be sure to clear the workspace before returning to the analysis folder and beginning analysis of another video.

## Data analysis

Videos were analyzed in MATLAB using a particle tracking routine, which finds the center of intensity for each microsphere using a polynomial Gaussian fit. Each 30-s video analyzed had a minimum of 10 trackable events. Videos were inspected to ensure that purely random motion and no drift were present, or drift was corrected in ImageJ (see step E1). Parameters within the particle tracking software were adjusted to only track singlets of microspheres, as doublets, triplets, and larger aggregates do not diffuse in the same manner that singlets do. Microspheres must also be at least 3 μm away from others to minimize hydrodynamic interactions. Further details of the data analysis method can be found in Lyon et al., Dudiak et al., and Rogers et al. [1,36,39]. The analyzed data demonstrate viscoelastic behavior of the mucus within the lumen of human gastric organoids and support functional studies of this mucus [36].

## Validation of protocol

This protocol (or parts of it) has been used and validated in the following research article:

- Lyon et al. [36]. Physical, chemical, and structural properties of human gastric organoid-derived mucus. *Am J Physiol Gastrointest Liver Physiol.*

## General notes and troubleshooting

**General notes**

1. Organoid selection: To ensure a consistent microenvironment and sufficient tracking volume, select only organoids with a diameter of at least 200 μm for injection that are free from cellular debris (Figure 1A). Smaller or more irregularly shaped organoids may be difficult to inject (Figure 1B, C). Organoids larger than 800 μm may be structurally unstable and experience luminal collapse upon injection [20]. Before proceeding with injection, it is recommended to visually confirm the approximate sizes of organoids with a digital microscope using a scale bar in the acquisition software.

2. System equilibration: Following injection, a 24-h incubation period at 37 °C and 5% CO_2_ is recommended to allow the microspheres to equilibrate within the mucus and for the organoid epithelium to recover from the mechanical stress of the capillary puncture. While imaging immediately after injection may be informative for different purposes, such a choice may yield results reflecting injection-induced flow rather than true passive diffusion.

**Troubleshooting**

**Problem 1:** Organoids rupture and expel luminal contents.

Possible cause: Mechanical stress from the injection needle or natural spontaneous rupture events where organoids periodically release their luminal contents (e.g., mucus, dead cell material) into the surrounding ECM [9].

Solution: Use additional time-lapse microscopy over the course of the entire experimental period to identify and exclude ruptured organoids from the experiment and subsequent dataset. Monitor the surrounding ECM for a diffuse fluorescent signal, which may indicate epithelial leakage (Figure 2A). Only proceed with analysis for organoids that maintain a stable, well-defined lumen throughout the duration of the imaging.

**Problem 2:** Microspheres appear as aggregates or clumps.

Possible cause: Insufficient vortexing of the stock solution.

Solution: Pulse-vortex the microsphere stock solution for at least 1 min immediately before each dilution and immediately before loading the injection capillary. If vortexing does not fix the problem, it is possible that a lower concentration of microspheres may be required for that particular application. The concentration may be modified as long as there are still enough trackable events for image analysis.

**Problem 3:** Difficulty visualizing organoids containing microspheres.

Possible cause: Injected organoids are located too deep within the ECM, exceeding the working distance of the selected objective.

Solution: Prioritize the injection of organoids positioned closest to the cover glass. Using a 40× or 60× objective can also mitigate this issue, but it can become more difficult to capture sufficient trackable events with a smaller field of view.

**Problem 4:** Electrostatic interactions causing microsphere immobilization.

Possible cause: Negative charges on gastric mucins can interact with the surface chemistry of carboxylated microspheres, leading to immobilization that is unrelated to the luminal mucus viscosity.

Solution: Consider using PEGylated microspheres to determine whether this is the issue and whether mucoadhesion may be reduced under these conditions. Compare results across different surface chemistries to control for electrostatic effects [41].

**Problem 5:** Lower moduli in microrheology data compared to bulk measurements.

Possible cause: Microrheology probes the local microenvironment and mesh size of the mucus, whereas bulk rheology measures the integrated response of the entire network. Gastric mucus is an inherently heterogeneous network. Tracer particles or microspheres exhibit probe-scale sampling of this network, with local mesh size sensitivity. At the microscale, these particles will display different deformation characteristics [33,42].

Solution: Ensure data are reported specifically as “microrheological” values and acknowledge this length scale sensitivity. When comparing to bulk measurements, use the Stokes–Einstein relation only for frequency ranges where the mucus behaves predictably (when stress and strain are proportional) [33]. As more knowledge is acquired about the expected mesh size of the mucus, the size of the chosen microspheres can be tailored accordingly for more precise measurement.

**Problem 6:** Optical artifacts.

Possible cause: Phase misalignment creating a “double image” of a single microsphere during bidirectional scanning.

Solution: While at the microscope, adjust the phase correction slide bar within the LASX acquisition software to merge any false doublets into a singlet. Given this step, post-processing for removal of doublets should not be necessary, and any doublets identified at this stage should not be counted as trackable events.

## Supplementary information

The following supporting information can be downloaded here:

1. Supplementary Table 1. Required MATLAB packages for microrheology particle tracking analysis.
